# Cardiac imaging findings in anomalous origin of the coronary arteries from the pulmonary artery; narrative review of the literature

**DOI:** 10.1186/s44156-022-00012-7

**Published:** 2022-12-06

**Authors:** Ali Ajam, Zahra Rahnamoun, Mohammad Sahebjam, Babak Sattartabar, Yasaman Razminia, Seyed Hossein Ahmadi Tafti, Kaveh Hosseini

**Affiliations:** 1 Tehran Heart Center, Cardiovascular Diseases Research Institute, Tehran University of Medical Sciences, Tehran, Iran; 2 Department of Echocardiography, Tehran Heart Center, Tehran University of Medical Sciences, Tehran, Iran; 3 Research Center for Advanced Technologies in Cardiovascular Medicine, Tehran Heart Center, Tehran University of Medical Sciences, Tehran, Iran; 4 Department of Cardiology, Tehran Heart Center, Tehran University of Medical Sciences, Tehran, Iran

**Keywords:** ARCAPA, Coronary artery anomaly, Right coronary artery

## Abstract

**Introduction:**

Anomalous origin of the right coronary artery from the pulmonary artery (ARCAPA) is a rare coronary artery malformation with an incidence of 0.002% in patients undergoing coronary angiography. It can lead to an increased risk of myocardial infarction (MI) and sudden cardiac death, even in asymptomatic patients.

**Methods:**

We conducted a review of published cases of ARCAPA using PubMed and Scopus databases and included patients over 18 years old with adequate echocardiographic data.

**Results:**

We evaluated 28 patients with ARCAPA with a mean age of 42.8 from 1979 to 2021. Patients were diagnosed mostly by angiography and echocardiography, the most performed treatment was reimplantation (15, 53.6%) and the main echocardiographic findings were dilated coronary arteries (9, 32.1%), coronary collaterals (8, 28.6%), and retrograde flow from right coronary arteries to main pulmonary trunk (7, 25%).

**Conclusion:**

Although ARCAPA is rare and not as deadly as the anomalous origin of the left coronary artery from the pulmonary artery (ALCAPA) still there is a chance of serious outcomes, therefore surgical treatment should be performed upon diagnosis. Angiography is the gold standard for diagnosis, but echocardiography can be a convenient, non-invasive, and most reliable method as the primary step whenever ARCAPA is suspected.

## Introduction

Anomalous origin of the right coronary artery from the pulmonary artery (ARCAPA) is the second most common type of coronary artery malformation in the category of abnormal origin of coronary arteries from the pulmonary artery with an incidence of 0.002% in patients undergoing coronary angiography, accounting for 0.12% of these anomalies [1, 2]. Despite being rare ARCAPA can lead to an increased risk of myocardial infarction and sudden cardiac death, even in asymptomatic patients, therefore a surgical correction is recommended when diagnosed. Echocardiography, multislice computed tomography angiography (CTA), and magnetic resonance angiography (MRA) are generally used to diagnose ARCAPA. Patients with ARCAPA are usually asymptomatic and may be incidentally detected when undergoing paraclinical examinations for other problems, a common example is a coronary angiography following chest pain [3, 4].

In this study, we reviewed cases of ARCAPA in recent years and the case we encountered in the Tehran Heart Center, focusing on echocardiographic findings.

## Methods

We conducted a review of published cases of ARCAPA using PubMed and Scopus databases in January 2022. The following keywords were used: “anomalous origin of the right coronary artery from the pulmonary artery”, “ARCAPA”, and “echocardiography”. We included case reports which described cases aged above 18 with the confirmed origin of the right coronary artery (RCA) from the pulmonary artery without other congenital cardiac anomalies. The reports should also include relevant information on patients’ echocardiographic examinations, especially signs of ARCAPA, and be published in English. The information on patients’ demographic and clinical data, method of diagnosis, and treatment were gathered when mentioned.

## Results

We identified 214 papers in our initial search (PubMed = 120, Scopus = 94) from 1978 to 2021. After screening based on title and abstract and removing duplicates, 34 papers were selected. Finally, 25 papers including 27 cases were eligible for our study after full-text reading [3–28]. The main characteristics of each case report are listed in Table 1. Patients had a mean age of 42.8 and 15 (53.6%) were women. Patients were diagnosed when being evaluated for cardiovascular symptoms ranging from a simple murmur (especially in younger patients) to severe chest pain and dyspnea suggesting myocardial infarction. Patients were diagnosed by coronary angiography (13, 46.4%), Echocardiography (7, 25.0%), CTA (6, 21.4%), and MRA (1, 3.6%). Reimplantation was the most performed procedure for treatment (15, 53.6%), 6 (21.4%) underwent coronary artery graft surgery (CABG), 2 (7.1%) had simple ligation, and 4 (14.3%) were treated without surgery.

**Table 1:** Literature review

Year	Author	Age/Sex	Presentation	Echocardiography	Diagnosis	Treatment
1979	Bermundez et al.	20/M	Chest Pain, Dyspnea	Dilated coronary arteries, Abnormal wall motion	Angiography	Reimplantation
1981	Salomon et al.	35/F	Dyspnea	LV dilation	Angiography	Ligation
1994	Dalström et al.	59/F	Chest Pain, Dyspnea	Normal LV size and function	Angiography	Conservative
1994	Veselka et al.	36/F	Chest Pain, Dyspnea	Dilated coronary arteries, RCA origin in PA	Echocardiography	Reimplantation
1996	Kautzner et al.	47/M	Chest Pain	Abnormal wall motion	Angiography	Reimplantation
1996	Kautzner et al.	36/F	Chest Pain, Dyspnea	Dilated coronary arteries, RCA origin in PA	Echocardiography	Reimplantation
2001	Endo et al.	30/M	Murmur	Normal LV size and function	Angiography	Reimplantation
2007	Su et al.	39/F	Chest Pain	Dilated coronary arteries, Retrograde flow	CMR	Reimplantation
2008	Park et al.	63/M	Chest Pain, Dyspnea	Normal LV size and function	MSCT	CABG
2011	Gilmour et al.	48/F	Chest Pain, Dyspnea	Dilated coronary arteries	Angiography	Reimplantation
2012	Altarabsheh et al.	34/M	-	Abnormal wall motion	MSCT	CABG
2012	Parasramka et al.	21/M	Chest Pain, Dyspnea	Abnormal wall motion	Angiography	Reimplantation
2013	Alfolabi-Brown et al.	55/F	Dyspnea	Coronary collaterals, Retrograde flow	Echocardiography	Conservative
2013	Courand et al.	19/F	Palpitation	Coronary collaterals, Retrograde flow	Echocardiography	Reimplantation
2013	Courand et al.	20/M	Loss of Conciseness	Coronary collaterals, Retrograde flow	Echocardiography	Reimplantation
2013	Maroules et al.	35/M	Murmur	Dilated coronary arteries	MSCT	-
2014	Nakabayashi et al.	44/M	Loss of Conciseness	Abnormal wall motion, LV dilation, RCA origin in PA	Angiography	Reimplantation
2017	Guzeltas et al.	18/M	Murmur	Coronary collaterals, Dilated coronary arteries	Echocardiography	Reimplantation
2017	Balakrishna et al.	55/F	Chest Pain	Normal LV size and function	Angiography	CABG
2018	Rawala et al.	57/M	Dyspnea	Abnormal wall motion, LV dilation	Angiography	CABG
2018	Farwati et al.	58/F	Chest Pain, Dyspnea	LV dilation	Angiography	Conservative
2019	Ganta et al.	41/F	Chest Pain	Coronary collaterals	-	Reimplantation
2019	VanLoozen et al.	57/F	Chest Pain	Abnormal wall motion, LV dilation	Angiography	Reimplantation
2020	Mantha et al.	53/F	Murmur	Normal LV size and function	MSCT	CABG
2020	Nishino et al.	58/F	Loss of Conciseness	Coronary collaterals, Retrograde flow	MSCT	Reimplantation
2021	Kappel et al.	76/F	Palpitation	Dilated coronary arteries, RCA origin in PA	Echocardiography	Conservative
2021	Teng et al.	18/M	Murmur	Coronary collaterals, Dilated coronary arteries	MSCT	Ligation
2022	Ajam et al.	66/M	Chest Pain	Retrograde flow	Angiography	CABG

As for echocardiographic findings, 8 (28.6%) had coronary collaterals, 9 (32.1%) had dilated coronary arteries and retrograde flow from RCA to pulmonary arteries was evident in 7 (25%). Signs of left ventricular dilation were present in 5 (17.9%) patients and 7 (25%) had abnormal wall motion. Echocardiography found RCA origin in PA in 4 (14.3%) of patients.

**Fig. 1:**
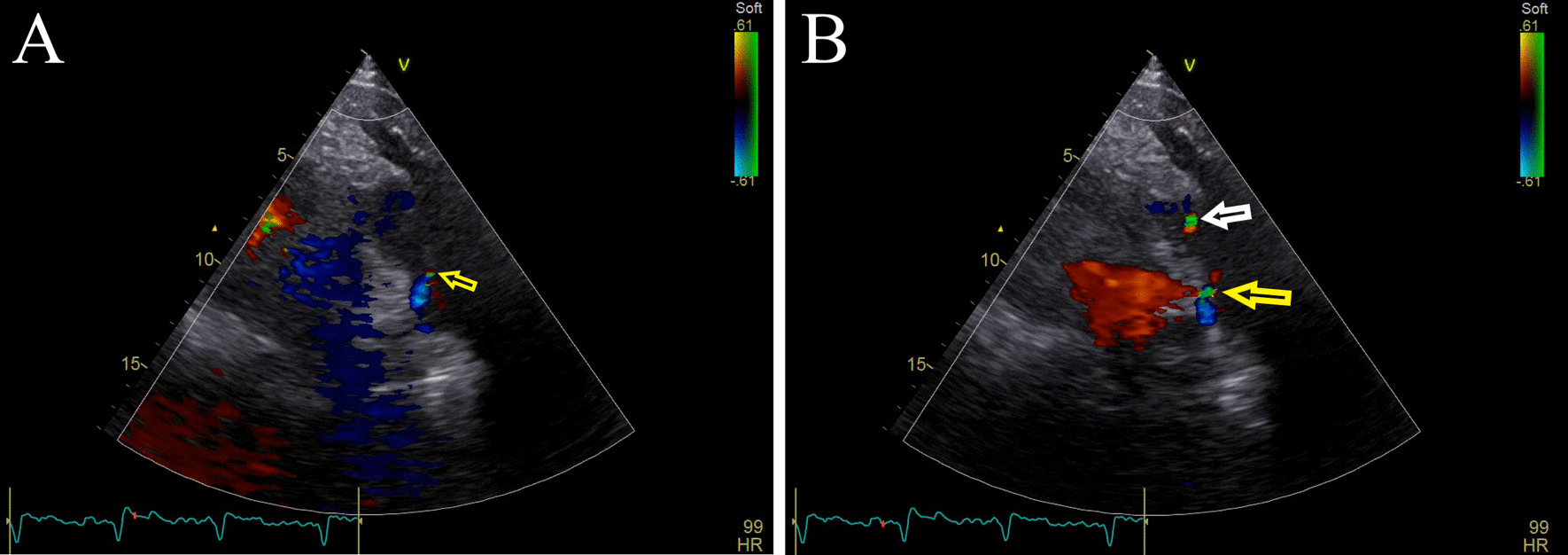
Echocardiography. **A** Parasternal right ventricular outflow view, abnormal flow during systole (yellow arrow). **B** Abnormal flow during diastole (yellow arrow) synchronous to pulmonary insufficiency jet (white arrow)

**Fig. 2:**
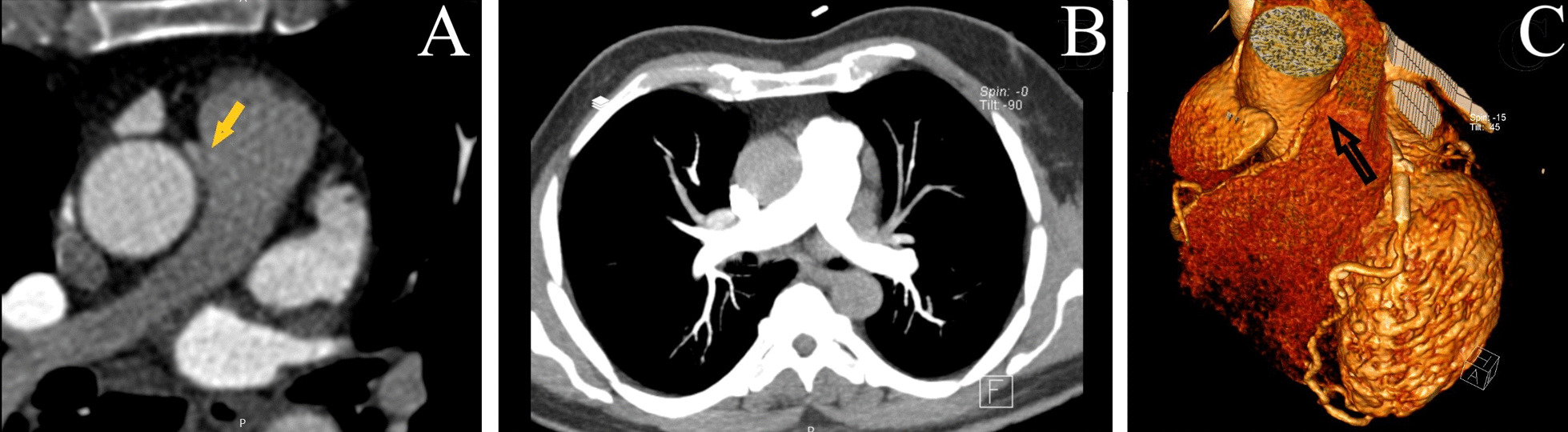
Computed Tomography Angiography. **A** Right coronary artery from the main pulmonary artery in the aortic phase. **B** No sign of right coronary artery in the pulmonary phase due to pressure gradient pre-operation. **C** Volume rendering technique (VRT), the origin of the right coronary artery from the main pulmonary artery

**Fig. 3:**
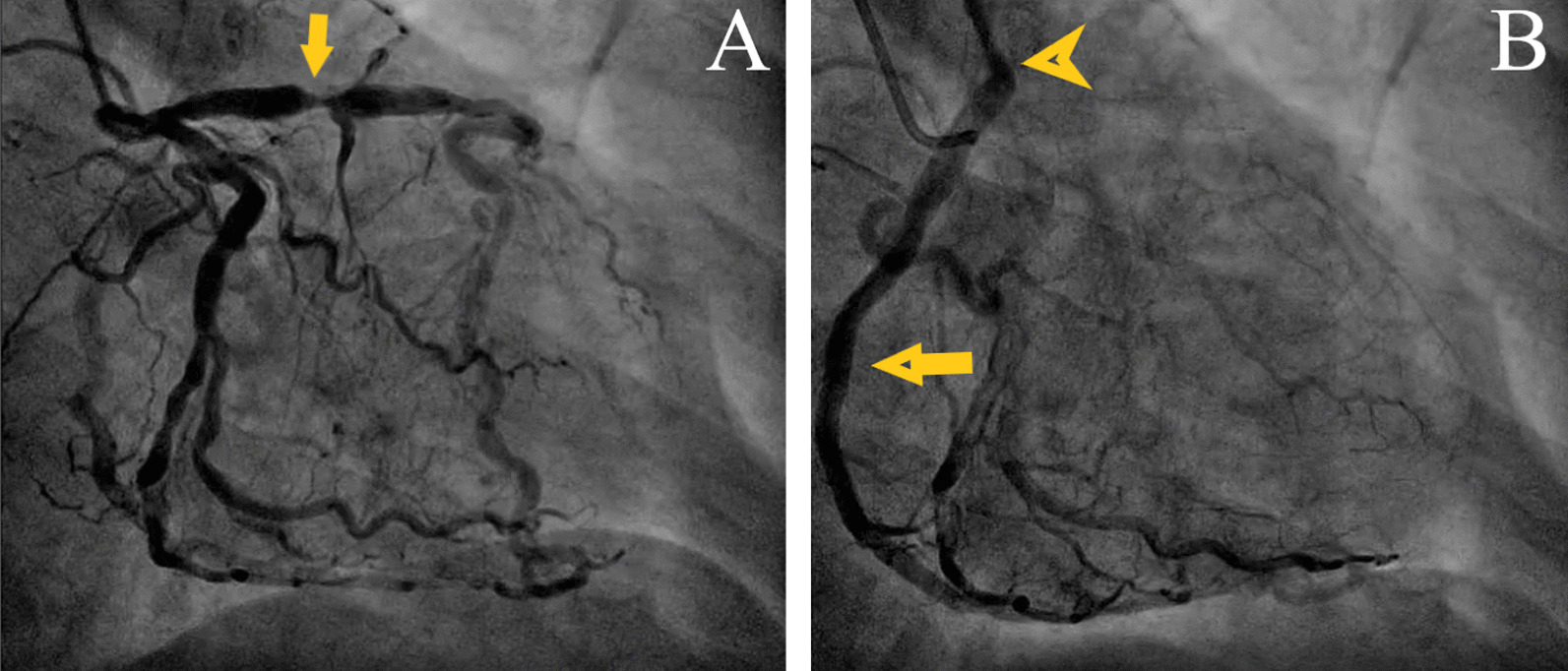
Angiography. **A** In-stent restenosis of the left descending artery (yellow arrow). **B** Origin of the right coronary artery from the main pulmonary artery

As for our case, a 66-year-old man presented to the emergency department of THC with the chief complaint of chest pain. He had a history of primary percutaneous catheter intervention (PCI) due to an anterior MI 2 years ago when ARCAPA was incidentally diagnosed. He underwent CABG surgery due to in-stent restenosis of the left descending artery (LAD). The left internal mammary artery was anastomosed to LAD and a saphenous vein graft to RCA. As RCA originated from the main pulmonary artery, the connection was ligated with a patch. The patient's paraclinical data are depicted in Figs. 1, 2, 3.

## Discussion

On the 12th day of life, coronary buds appear after the truncus arteriosus begin to divide by spiral septum forming the aorta and pulmonary artery. Mal-positioning of coronary buds or malrotation of the spiral septum can lead to coronary artery anomalies [29]. In a study on a series of 38,000 patients undergoing cardiac catheterization, coronary artery anomalies were detected in 1.6%. Congenital coronary artery anomalies are divided into 4 groups, anomalous origin of a coronary artery from the pulmonary artery, the ectopic origin of a coronary artery from the aortic sinus, absence of a coronary artery, and coronary artery fistula [30]. The most common type of anomalous coronary artery with connection to the pulmonary artery is the anomalous origin of a left coronary artery from the pulmonary artery (ALCAPA) with an incidence of 0.008% followed by ARCAPA (0.002%). Due to the higher oxygen demand of the left ventricle and bigger territory fed by the left coronary artery (LCA) symptoms appear early in life leading to early diagnosis, Myocardial ischemia and sudden cardiac death are also more common in ALCAPA for the same reason [29, 31].

Since 1882 when John Brooks described the first case of ARCAPA, more than 100 cases of ARCAPA are identified. Patients with ARCAPA are usually asymptomatic and usually detected incidentally when the patient is undergoing cardiac evaluation for other problems. But when symptomatic, although uncommon, can include dyspnea, fatigue, congestive heart failure, angina, MI, and even sudden cardiac death [26]. ARCAPA symptoms onset has two peaks, one early after birth and another between 40 and 60 years. Early in life, high pulmonary pressure feeds RCA territory with deoxygenated blood, leading to the formation of collateral vessels. When pulmonary pressure drops, these collaterals result in the backflow of oxygenated blood from the LCA system to the pulmonary artery which is called “coronary steal”. If this phenomenon happens, symptoms of ischemia appear in the early years. As for later onset symptoms, because the single functioning coronary system is more prone to atherosclerosis, ARCAPA patients develop ischemia around 40–60 [6].

Before 1965 all ARCAPA cases were diagnosed during surgery or at autopsy but due to cardiac imaging advancement coronary angiography and echocardiography are the most popular methods nowadays [14]. Cardiac angiography is still the gold standard method for coronary artery anomalies, despite being invasive it can establish a complete map of coronary vasculature which can also be helpful for the treatment of anomalies. Retrograde flow through the RCA, increased flow with dilation of the LCA/RCA, direct flow between the pulmonary artery and the RCA, and collateralization between the left and RCA systems are features suggestive of ARCAPA in angiography [32].

Transthoracic echocardiography (TTE) is a safe, non-invasive, inexpensive, and portable method to examine the origin of coronary arteries [1]. Echocardiography also has the chance of finding synchronous cardiac lesions, which was seen in 23.8% of ARCAPA patients. knowledge of these lesions can improve myocardial protection strategies when performing cardiopulmonary bypass, reducing post-operative complications [32].

One of the best views to visualize the origins of coronary arteries is the parasternal short-axis. In patients with ALCAPA, there are usually signs of myocardial ischemia. Endocardium and papillary muscles look “shiny”. Reverse flow from the myocardium to the pulmonary trunk is one of the most important findings suggesting the diagnosis. in addition, as the lesion is a fistula, and due to increased collateral flow from the right to the left coronary system, there is an increase in flow across minor coronary arteries which is detected by lowering the Nyquist limit. It is essential to perform color flow mapping because isolated cross-sectional imaging may find LCA a normal aortic origin. After all, sometimes LCA takes an intramural course through the aortic wall in ALCAPA. In the younger patient, the most striking features are dilation of the RCA and collateral arterial flow in the ventricular septum which can be misdiagnosed as multiple muscular ventricular septal defects (VSD). To distinct these two, pulse-wave interrogation should be performed. ALCAPA has continuous flow in collateral arteries, whereas in VSD cases the major finding is systolic color flow entering the right ventricle [28, 33].

ARCAPA is easier to find in echocardiography compared to ALCAPA. Main findings in adult patients with ARCAPA include intraseptal coronary collaterals in using color flow Doppler mapping, dilation of coronary arteries, increased flow in the LCA, and flow between the RCA and the pulmonary artery. Sometimes even the ostium of RCA can be seen entering the pulmonary artery trunk. The left coronary artery is usually enlarged and tortuous and collateral arteries are present in the ventricular septum as seen in ALCAPA. Wall motion abnormalities in ventricles can also be seen in TTE [4, 6, 10–14, 28, 32]. When there is a dilated LCA and continuous or diastolic retrograde flow from an anomalous vessel to the pulmonary artery, ARCAPA can be mistaken for a coronary fistula. It is usually the case when the origin of ARCAPA is not visualized. In a real coronary fistula, the origin of RCA can be traced back to the right sinus of Valsalva. Atresia of RCA is also a differential diagnosis when dilated LCA is present with inter-coronary collaterals in the ventricular septum. In this case, the origin of RCA from pulmonary should not be visible [1].

Transesophageal echocardiography also further distinguishes the tortuous anomalous coronary arteries and characterizes their flow patterns. It has the advantage of the proximity of the probe to the interest area, in addition to better spatial resolution due to a higher frequency transducer. This method also has some limitations because vessels have a curvilinear path along the epicardial surface, and it is very difficult and time-consuming to visualize their entire course [1].

It is crucial to mention that even with a detailed echocardiographic examination the diagnosis can be easily missed because there are inherent limitations in ultrasound resolution which can lead to difficulty distinguishing the origin of coronary arteries, especially because there can be an ultrasonic dropout in the wall of the aorta near transverse sinus and left coronary artery. Therefore, TTE can only be used to rule in the diagnosis not ruling out. Albeit Doppler color flow improved this diagnosis significantly. It is recommended that when the coronary artery appears to originate from the aorta in echocardiography but all the facts point to anomalous origin from the pulmonary trunk, a different modality should be used [33]. CTA and MRA can provide a 3-dimensional representation of cardiac anatomy, helping confirm the suspected diagnoses of echocardiography. MRA has the advantage of determining blood flow direction and velocity as well as not requiring ionizing radiation or intravenous contrast [14]. The main disadvantage of these methods is their high price, which highlights echocardiography's role as a quick first step when ARCAPA is suspected [1].

Although most cases of ARCAPA have isolated lesions, approximately 40% of patients have concomitant congenital cardiac defects. Aortopulmonary window is the most common associated congenital lesion with ARCAPA accounting for one-fourth of cases [1, 3, 6, 14, 34]. Other associated lesions include VSD, atrial septal defect (ASD), tetralogy of Fallot, patent ductus arteriosus, aberrant right subclavian artery, and bicuspid aortic valve [32, 34]. The aortopulmonary window is characterized by abnormal communication between the pulmonary artery and ascending aorta with two separate semilunar valves. When it is associated with ARCAPA, the pulmonary artery pressure is kept high therefore the flow in the anomalous coronary artery is maintained and myocardial ischemia usually doesn’t develop. Moreover, there is no sign of dilation or tortuosity in coronary arteries and collateral circulation in contrast to isolated ARCAPA in echocardiography [35, 36].

Nearly all studies suggest surgical treatment as soon as ARCAPA is diagnosed due to the chance of myocardial ischemia and cardiac failure. Patients who were not surgically treated had shorter life spans, and besides surgical correction have low postoperative mortality of 2–3%. Surgical treatment of ARCAPA has 2 purposes, elimination of left to right shunt or “coronary steal” and establishment of dual coronary circulation from the aorta [3, 6]. Formerly, ligation of the anomalous coronary artery was often used, and oxygenation of RCA territory was dependent on the collateral vessels, but this method was associated with high early and late mortality [33]. Now there are 3 surgical options for ARCAPA treatment, reimplantation of the anomalous coronary artery to the ascending aorta, ligation of ARCAPA, and CABG using saphenous vein graft, and single ligation. Reimplantation is the ideal one because it can achieve both treatment goals. CABG is selected when ARCAPA originates from the posterior wall of the pulmonary artery and reimplantation cannot be done. Ligation should be the last option when CABG and reimplantation are not possible [2].

### Limitations

There are several limitations to our study. First, we only included published ARCAPA cases, and thus unreported cases were not analyzed. Second, echocardiographic findings are highly operator dependent and we were confined by what the authors report in their examinations. Third, comprised cases were gathered over nearly 40 years and echocardiographic devices and diagnostic criteria have highly evolved, leading to disparity in findings. Albeit, we used recent suggested echocardiographic findings in ARCAPA for comparison and removed those cases that did not match our criteria.

## Conclusion

In conclusion, although ARCAPA is rare and not as deadly as ALCAPA still there is a chance of serious outcomes, such as MI and sudden cardiac arrest, therefore surgical treatment should be performed upon diagnosis. It should also be mentioned that although angiography is the gold standard for diagnosis, echocardiography can be a convenient and noninvasive method whenever ARCAPA is suspected.
